# A Data Set of Human Body Movements for Physical Rehabilitation Exercises

**DOI:** 10.3390/data3010002

**Published:** 2018-01-11

**Authors:** Aleksandar Vakanski, Hyung-pil Jun, David Paul, Russell Baker

**Affiliations:** 1 University of Idaho, Industrial Technology, 1776 Science Center Drive, Idaho Falls, ID 83402, USA; 2 University of Idaho, Department of Movement Sciences, 875 Perimeter Drive, Moscow, ID 83844, USA

**Keywords:** movement data set, physical therapy exercises, physical rehabilitation, motion capturing, human skeletons

## Abstract

The article presents University of Idaho – Physical Rehabilitation Movement Data (UI-PRMD) — a publically available data set of movements related to common exercises performed by patients in physical rehabilitation programs. For the data collection, 10 healthy subjects performed 10 repetitions of different physical therapy movements, with a Vicon optical tracker and a Microsoft Kinect sensor used for the motion capturing. The data are in a format that includes positions and angles of full-body joints. The objective of the data set is to provide a basis for mathematical modeling of therapy movements, as well as for establishing performance metrics for evaluation of patient consistency in executing the prescribed rehabilitation exercises.

## 1. Summary

Patient participation in physical therapy and rehabilitation programs is an important step in the recovery process of various musculoskeletal conditions. A home exercise program (HEP), where patients perform a set of recommended physical exercises in a home-based environment, is often a substantial component of a patient’s rehabilitation treatment. Despite the enormous expenses incurred by therapy programs on behalf of both healthcare providers and patients [1], HEP treatment is not necessarily successful in aiding the patient reach full functional recovery [2]. Reports in the literature indicate that one of the main barriers to successful HEP implementation is patient noncompliance with the prescribed exercise plan [3]. The low adherence rates are attributed primarily to the lack of supervision and monitoring of patient performance in the outpatient setting by a rehabilitation professional. In addition, psychological issues associated with fear of painful movements, fear of re-injury, and anxiety of increased pain have also been reported as important barriers to adherence in unsupervised exercise programs [4, 5]. Subsequently, a body of work has been concentrated on the development of tools in support of HEP, such as robotic assistive devices [6], exoskeletons, haptic devices, and virtual gaming environments [7].

The authors of this article are currently participating in a research project focused on the development of a novel therapy-supporting tool for automated monitoring and evaluation of HEP episodes based on implementation of machine learning algorithms. The goal of the project is to employ a Microsoft’s Kinect sensor [8] for capturing body movements during therapy sessions, and automatically evaluate patient performance and adherence to the recommended exercises. To achieve this goal, the project encompasses several specific objectives related to the development of a methodology for mathematical modeling of patient movements, definition of performance metrics, and creation of a set of therapy movements. The authors have developed a preliminary model of human motions using a machine learning method based on a neural network architecture consisting of auto-encoder and mixture density sub-nets [9], and they have proposed a taxonomy for performance metrics for evaluation of therapy movements [10]. The publications [9, 10] do not use the UI-PRMD set that is presented here. These articles use the data related to general human movements from the University of Dallas at Texas (UTD) - MHAD set [11], to prove the proposed concepts in a lack of a data set of therapy movements.

Machine learning methods employ observed or measured data of a system/process for establishing the relationship between the input and output parameters. These methods typically require vast amounts of data for learning the relationships among the system parameters. For many problems in the field of machine learning, the performance of the algorithms and their ability to extract relevant and useful information from data are directly proportional to the quantity and quality of the available data. In recent years, the research community in machine learning has become increasingly aware that the provision of appropriate data sets for a specific problem is essential for enhanced performance of the existing algorithms, and for developing and evaluating new algorithms. Moreover, at the present time companies that possess large data bases are often considered to have competitive advantage and capacity to achieve better results over other companies that work on a same problem. Consequently, a great deal of recent research efforts has concentrated on the creation of data sets for various problems in machine learning [12].

Our main motivation for creating the presented data set was the identified lack of publically available comprehensive data sets of physical therapy movements. Currently, there are a large number of publically available data sets related to general human movements [13] that are extensively used for tasks like action recognition, gesture recognition, pose estimation, or fall detection. Many of these data sets employ optical motion capturing systems for recording the movements, e.g., CMU Multi-Modal Activity (CMU-MMAC) [14], and Berkley MHAD (Multi-Modal Human Action Dataset) [15]. Likewise, numerous data sets of general human movements have been created by using the Microsoft Kinect sensor, such as the MSR (Microsoft Research) Action3D data set [16] and the previously mentioned University of Dallas at Texas - MHAD set [11].

The existing data sets of therapy movements, however, are limited either in the scope of the movements or in the provided data format. One such example is the HPTE (Home-based Physical Therapy Exercises) data set of therapy movements, created by Ar and Akgul [17]. The set contains eight shoulder and knee exercise movements performed six times by five subjects, recorded with a Kinect camera. One major limitation of the HPTE data set is that only the video and depth streams from the Kinect sensor are provided. The data set does not provides the corresponding body joint positions or angles, and although it is possible to extract the joint information from the video and depth frames, it is not a straightforward task, and it would require implementation of an image processing method. The EmoPain data set [4] was designed with an emphasis on studying pain-related emotions in physical rehabilitation, and contains high-resolution face videos, audio files, full body joint motions, and electromyographic signals from back muscles. A group of 22 patients and 28 healthy control subjects performed 7 exercises typically undertaken by patients with chronic lower back pain. Another data set presented in the work of Nishiwaki *et al*. [18] is restricted to three exercises of lower limbs performed by nine subjects. The activity of four leg muscles was recorded with EMG (electromyography) electrodes. In addition, several related data sets focus on physical activity monitoring (e.g., by wearing heart rate monitors, inertial measurement units [19]), and are typically applied for recognition or classification of the type of activity based on collected data.

The UI-PRMD set presented in this work includes 10 movements that are commonly completed by patients in physical therapy programs. A sample of 10 healthy individuals repeated each movement 10 times in front of two sensory systems for motion capturing: a Vicon optical tracker, and a Kinect camera. The movement data were collected in the Integrated Sports Medicine Movement Analysis Laboratory (ISMMAL) with the Department of Movement Sciences at the University of Idaho. The movement data have been categorized, organized and posted on a dedicated web site for a free public access. Potential benefits of publically posting the UI-PRMD set include the potential to serve as a benchmark for comparison of future research in physical therapy, and to streamline the process of establishing consistent metrics for evaluation of patient progress in rehabilitation.

The presented data set does not consider the implication on the selected movements by a particular type of injuries of level of injuries. As stated earlier, the goal of the research at this stage is to employ the data for mathematical modeling of rehabilitation movements in general, and for assessment of the deviation of motion trajectories from the derived movement models.

The organization and format of the data, as well as the movement type and files nomenclature are presented in Section 2. Section 3 describes the methods for recording the data, and provides information related to the order of the joint positions and angular displacements for the used sensory systems. Section 4 presents concluding remarks related to the contribution and limitations of the data set.

## 2. Data Description

**Figure 1:**
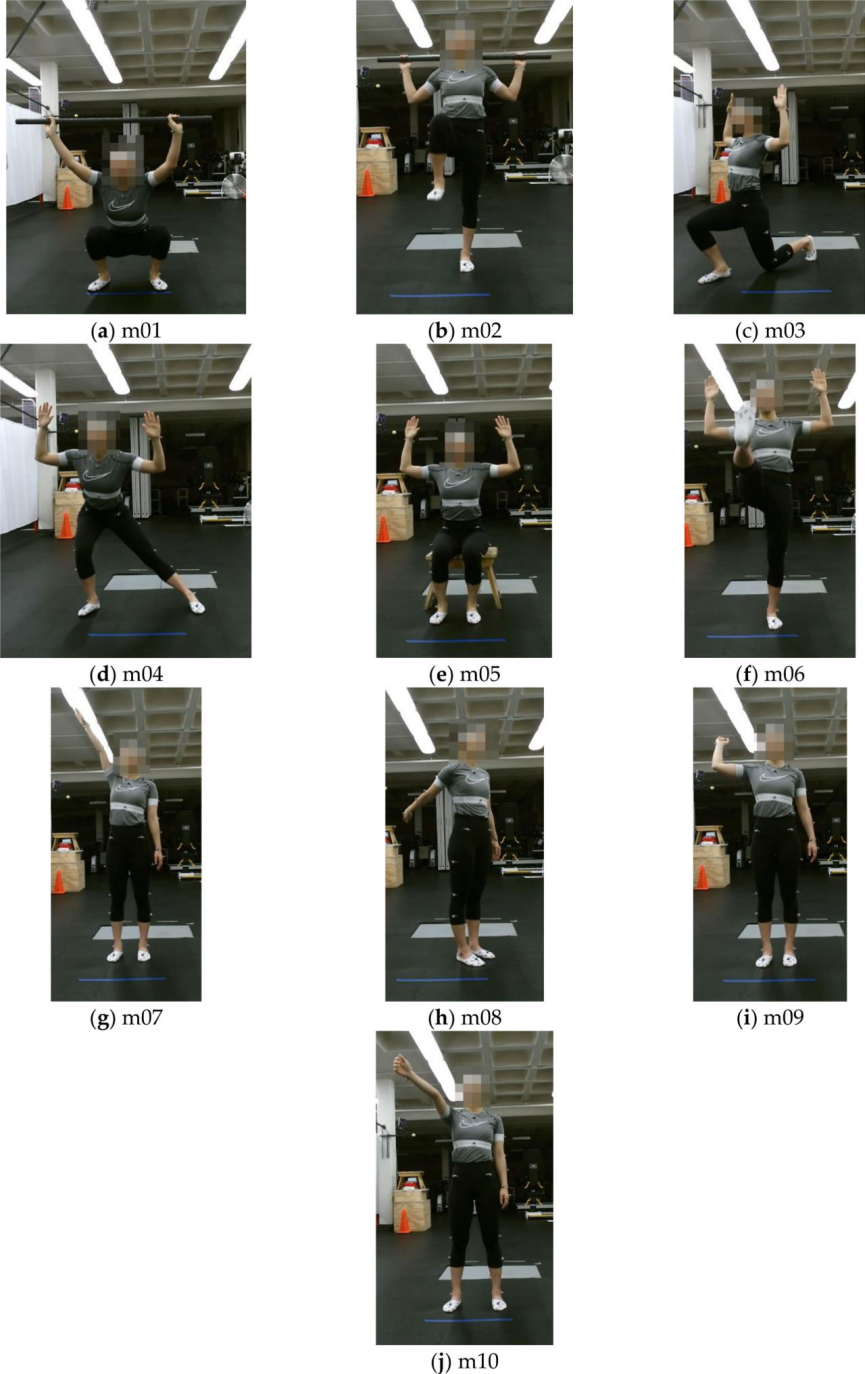
Examples of the 10 movements: (**a**) Deep squat (m01); (**b**) Hurdle step (m02); (**c**) Inline lunge (m03); (**d**) Side lunge (m04); (**e**) Sit to stand (m05); (**f**) Standing active straight leg raise (m06); (**g**) Standing shoulder abduction (m07); (**h**) Standing shoulder extension (m08); (**i**) Standing shoulder internal-external rotation (m09); (**j**) Standing shoulder scaption (m10).

The UI-PRMD data set consists of measurements of joint angles and positions of 10 subjects while performing movements commonly performed during physical rehabilitation exercises. We selected 10 different movements, which are listed and described in Table 1. Examples of the 10 movements are displayed in Figure 1. During the data collection, each movement was initially demonstrated to a subject by one of the authors of the study, and afterwards the subject was asked to perform multiple repetitions of the movement. Each subject performed 10 repetitions of each of the 10 movements. The subjects were not asked to maintain the body posture at the end of the repetitions for a period of time.

One motivation for selecting these movements is the body of work in the literature that, similarly to our research goal, dealt with modeling and evaluation of rehabilitation exercises. For instance, Lin and Kulic [20] employed a data set consisting of deep squats, sit to stand, knee flexion, hip flexion and straight leg raise movements for the development of a machine learning method for automated segmentation of the repetitions in each exercise. Komatireddy et al. [21] proposed an approach for evaluation of the consistency in completing the following physical therapy exercises: deep squats, inline lunge, sitting knee extension, and standing knee extension. Similar movements were employed in other related research within the published literature [17, 18]. Additional motivation for selecting the movements is because they are commonly used by clinicians as part of rehabilitation programs or as part of physical examinations for numerous situations, such as post-surgery recovery, upper body conditions (e.g., rotator cuff tendinopathy), lower body conditions (e.g., patellar tendinopathy) [22, 23]. On the other hand, the choice of the movements in the presented data set was not intended to address rehabilitation for specific types of medical or musculoskeletal conditions. Furthermore, the subject performance in the data set was not expected to correspond to a perfect movement, e.g., from a sport perspective. Rather, the goal was to collect motion trajectories that are performed in a consistent manner by the group of healthy subjects, and to utilize the data for mathematical modeling and analysis of the movements.

The nomenclature of the files in the data set includes the following information:

- Movement number _ subject number _ positions/angles
  For example, the data instance ‘m04_s06_positions’ pertains to the 4^th^ movement in Table 1 (i.e., side lunge) performed by the 6^th^ subject, and it consists of the Cartesian position coordinates of the body joints, expressed in millimeters. Similarly, ‘m08_s02_angles’ corresponds to the recorded angular displacements for the 2^nd^ subject while performing the standing shoulder extension movement, expressed in degrees. The joint order and further description of the position and angular measurements are provided in the next section, which is dedicated to the description of the data recording method.

The data are presented in ASCII txt format, with comma delimiter used for separating the data values in the files.

The data are organized into two folders ‘Vicon’ and ‘Kinect,’ each containing the measurements acquired by either of the two respective sensory systems. Each folder contains two subfolders, ‘Positions’ and ‘Angles,’ which contain the files with the respective measurements.

Further, because each movement consists of 10 episodes, or repetitions of the same movement, the data are also provided in a segmented form, where each file comprises the measurements for one episode of one of the movements. The corresponding data are provided in the ‘Segmented Movements’ folder. The following file nomenclature is used for the segmented movements:

- Movement number _ subject number _ episode number _ positions/angles
  In this case, the file ‘m04_s06_e10_angles’ consists of the angular joint measurements for the 10^th^ episode of the 4^th^ movement performed by the 6^th^ subject.

In addition, the data set provides examples of the movements performed in an incorrect, or non-optimal, manner. In Table 1, the rightmost column provides explanations of non-optimal performance for each movement. For instance, incorrect ways to perform the deep squat movement can include: upper torso is not kept vertical during the squat, knees are not aligned and kept parallel, noticeable or excessive trunk flexion, or noted loss of balance, among. The rationale for the inclusion of non-optimal performance is that the correctly performed movements are associated with examples of movements that are demonstrated to a patient by a rehabilitation professional, and therefore they can be utilized to develop a mathematical model of the movement. In contrast, patients with musculoskeletal injury or constraints are assumed to be unable to, at least initially, perform the exercise movement in a correct or optimal manner that is generally accepted as necessary for efficient movement to reduce injury risk associated with physical activity [24]. Consequently, the consistency of the incorrectly performed movements can be evaluated in relation to the derived mathematical model of the correct movements, and a performance score can be communicated to the patient by an automated system for movement analysis. In other words, the non-optimal portion of the data can serve as a testing set, and it can be used for validation of the formulated mathematical models for the correct portion of the movements.

In creating the set of incorrect movements, the subjects performed 10 episodes of the 10 movements arbitrarily in a suboptimal manner. One should note that the subjects were not asked to simulate a patient with a specific injury, nor to perform the incorrect exercises at a certain level of a specific injury. The goal was to execute a set of non-optimal movements that represent deviation from the correctly executed movements. This portion of the data is stored into a folder named Incorrect Movements. Analogously to the described taxonomy for the correct movements, the data are classified based on the sensory system used (Vicon or Kinect), and based on whether the movements are available in their entirety or segmented into 10 episodes. The file nomenclature for the incorrect movements follows the same order as for the correct movements, and in addition, all files have an extension _inc, which implies ‘incorrect.’ For instance, the file ‘m05_s04_e03_angles_inc’ corresponds to the incorrect 3^rd^ episode performed by the 4^th^ subject for the sit to stand movement. One of our next goals and a future work task is to manually label the incorrect movements, based on the extent of inconsistency in performing the exercises.

The files m03_s03_positions/angles for the Vicon system, related to the inline lunge movement performed by the 3rd subject, are missing in the data set, due to absent measurements for the markers during the data collection. The corresponding data for the same movements recorded with the Kinect sensor is available in the data set.

## 3. Methods

The data were collected using two types of sensory systems: a Vicon optical tracking system, and a Microsoft Kinect camera.

Vicon optical tracker [25] is a highly accurate system designed for human motion capturing and analysis. The system employs eight cameras with high speed and resolution characteristics for tracking a set of retroreflective markers. By attaching the set of markers on strategic locations of a human body, the system calculates the position of the markers based on the acquired data from the cameras, and it uses this information to retrieve the orientations of the individual body parts.

The Kinect sensor [8] consists of a color camera and an infrared camera, which are used to simultaneously acquire image and range data from the environment. The sensor was initially designed as a natural-user interface in gaming environments for Microsoft’s Xbox console; however due to its popularity among researchers, hobbyists, and the industry, Microsoft offered it as a stand-alone unit for Windows operating systems and released a software development kit (SDK). The SDK provides libraries for access to the raw RGB and depth streams, other miscellaneous data processing codes, and the most importantly for this study — a skeletal tracker with real-time motion capturing ability. In skeletal mode, the Kinect sensor can track the movements of up to six people and 25 skeletal joints per person.

The movements performed by the study participants were acquired simultaneously with the Vicon and Kinect systems. The software programs Nexus 2 and Brekel were employed for recording the movements with the Vicon and Kinect systems, respectively. The frame rate of the motion capture with Vicon was 100 Hz, whereas for the Kinect it was 30 Hz. The Cartesian positions values for the joints are expressed in millimeters, and the joint angles are expressed in degrees, for both the Vicon and Kinect measurements. The values in the data set are presented as recorded. The only preprocessing operation that was performed corrected large jumps in the angle measurements with Kinect, because the angles were limited to be in the (−180°, +180°) range. For the cases where these limits were exceeded, the values continued on the opposite side of the limits. No other data processing was performed.

**Figure 2:**
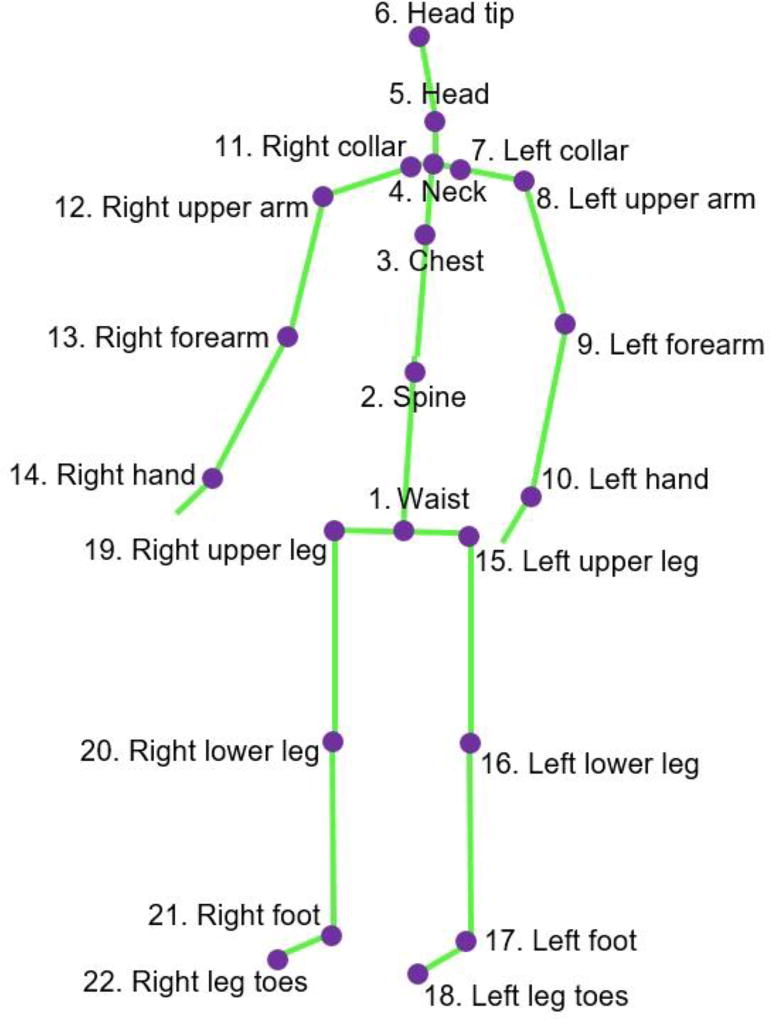
Joints in the skeletal model of Kinect recorded data.

The joints in the skeletal model recorded with the Kinect sensor are shown in Figure 2. The data include the motion measurement for 22 joints. The positions of the fingers are not included because they are not relevant for assessing correct performance of the movements included in the study. The order of the joint measurements in the data set is displayed in the figure, where the first three measurements pertain to the waist joint, the next three values are for the spine, etc. The values for the waist joint are given in absolute coordinates with respect to the frame of the coordinate origin of the Kinect sensor, and the values for the other 21 joints are given in relative coordinates with respect to the parent joint in the skeletal model. For instance, the position and orientation of the left forearm is given relative to the position and orientation of the left upper arm.

**Figure 3:**
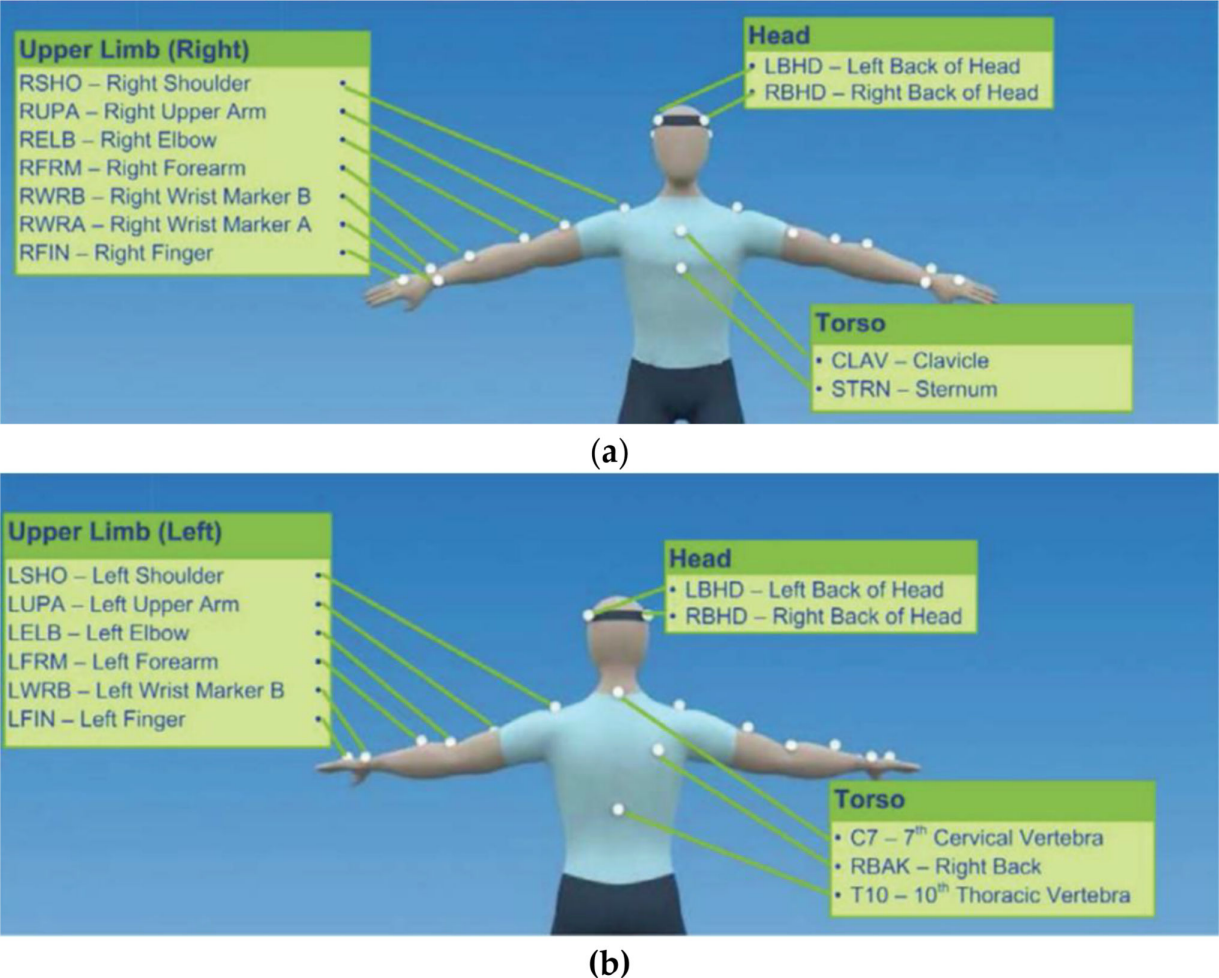

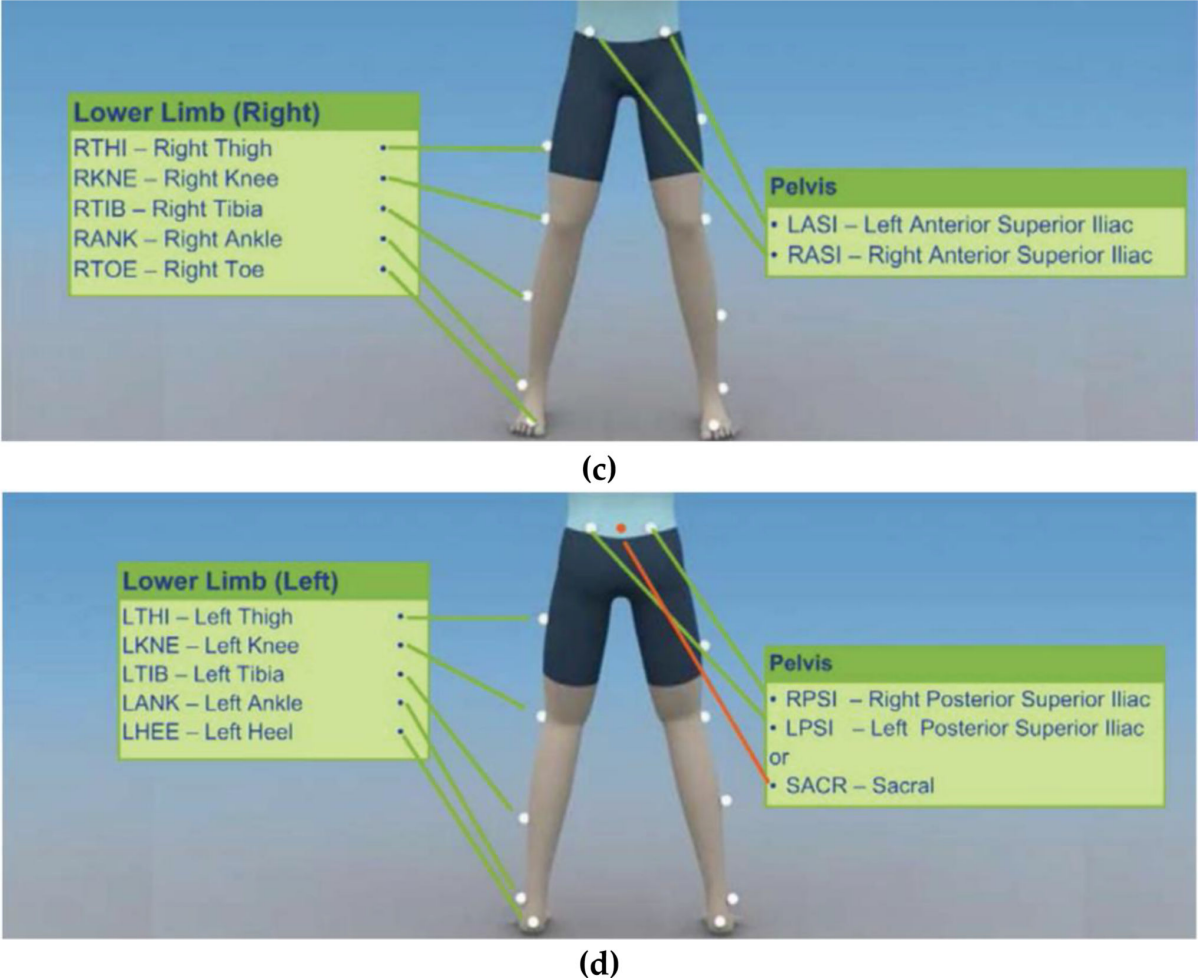
Locations on the body for attaching the Vicon markers. (**a**) Front view of the upper body; (**b**) Back view of the upper body; (**c**) Front view of the lower body; (**d**) Back view of the lower body. The pictures are taken from [26]. Copyright: © 2016 Vicon Motion Systems Limited.

For tracking and sensing of the demonstrated movements with the Vicon system, a total of 39 reflective markers were attached on the subjects’ bodies. The locations for attaching the markers [26] are shown in Figure 3.

The order of measurements for the Vicon and Kinect systems are presented in Table 2. For both Vicon and Kinect, the joints for which the measurement are absolute are given with respect to the coordinate system of the sensory system, and are indicated in the parenthesis in the table. For the remaining joints, the measurements are relative, and are given with respect to the parent joint in the skeletal model. The angle outputs for all joints are represented with the YXZ triplet of Euler angles for both sensors.

**Figure 4:**
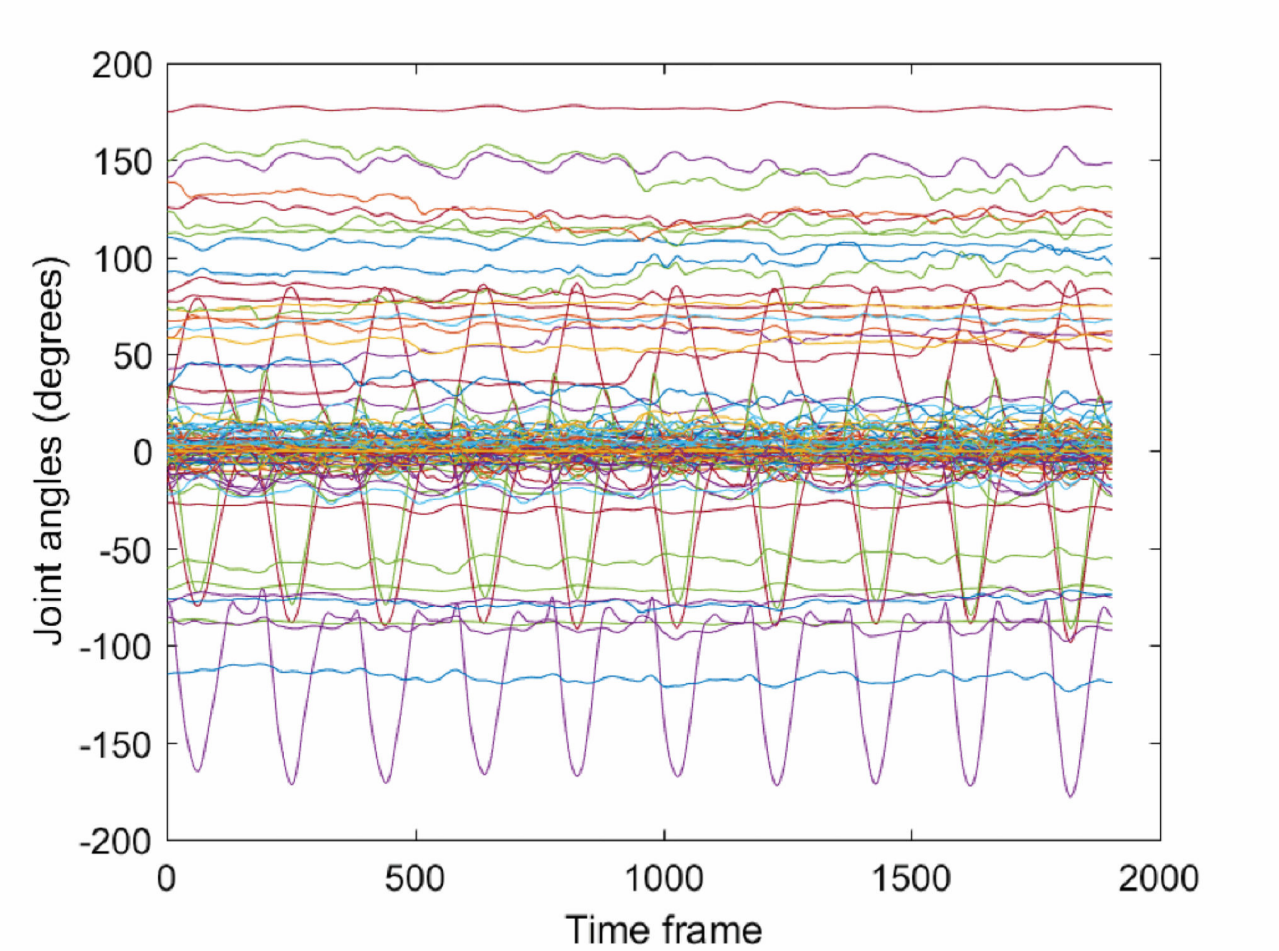
Recorded joint angles with the Vicon motion capture system for one subject showing the 10 episodes of the standing active straight leg raise movement. The figure displays the angular displacements corresponding to the 117-dimensional data for approximately 1,900 time frames (i.e., 19 seconds).

A sample of the collected data with Vicon for the m06_s01_angles file, which is related to the standing active straight leg raise movement, is shown in Figure 4. All angular coordinates for the 39 joints are given in the figure in degrees. One can notice that only several joints have significant displacements and they correspond to the leg joints, whereas the other body joints have almost constant values.

The demographic information of the ten subjects who participated in the data collection is provided in Table 3. The average age of the subjects was 29.3 years, with the standard deviation of 5.85 years. As stated before all the subjects were in a good health condition. The subjects were either graduate students or faculty at the University of Idaho, as indicated in the table.

In spite of the provided information for the dominant side of the subjects in Table 3, some of the subjects were not consistent in performing the exercises with their dominant hand or leg. Table 4 lists the hand or leg with which each subject performed each of the movements, where understandably, R and L stand for right and left, respectively. The deep squat and sit to stand movements are not listed in the table because they don’t depend on the subjects’ dominant side.

**Figure 5:**
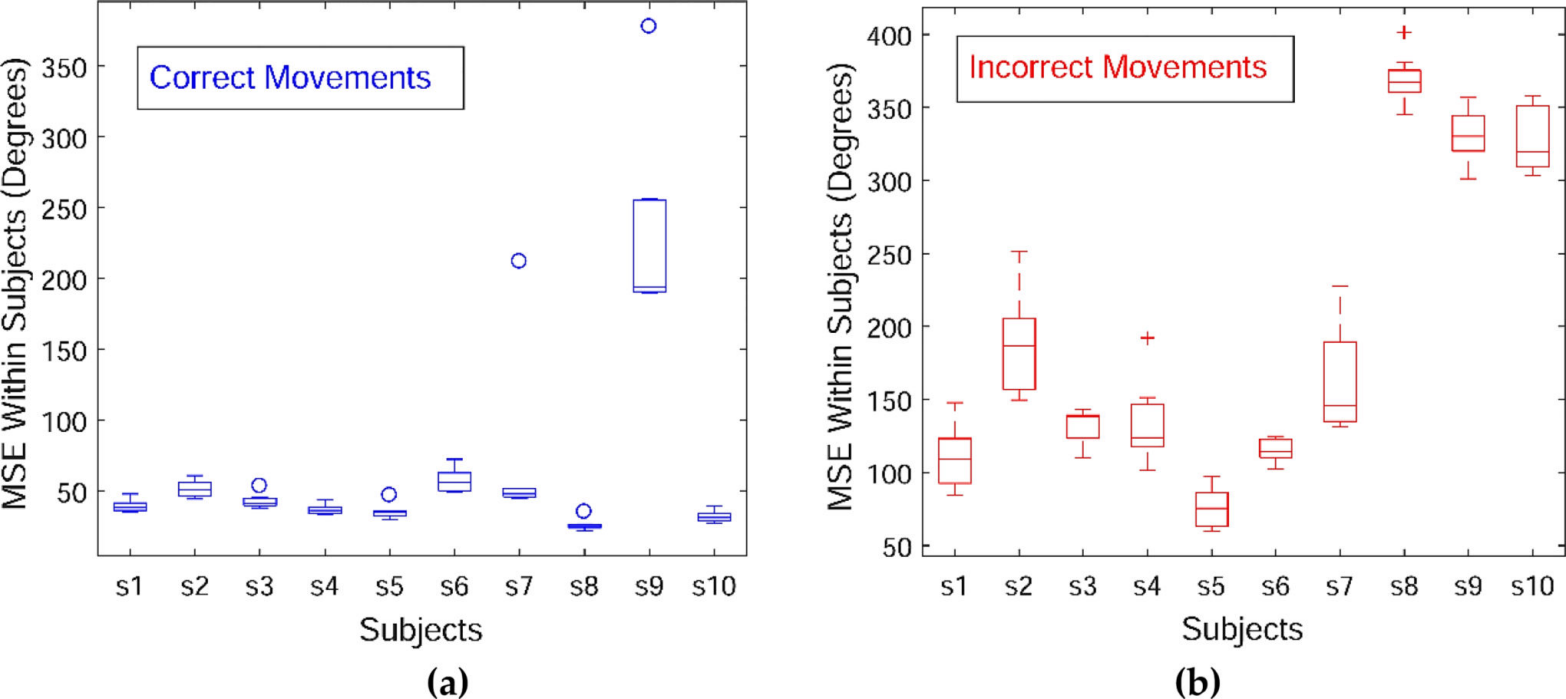

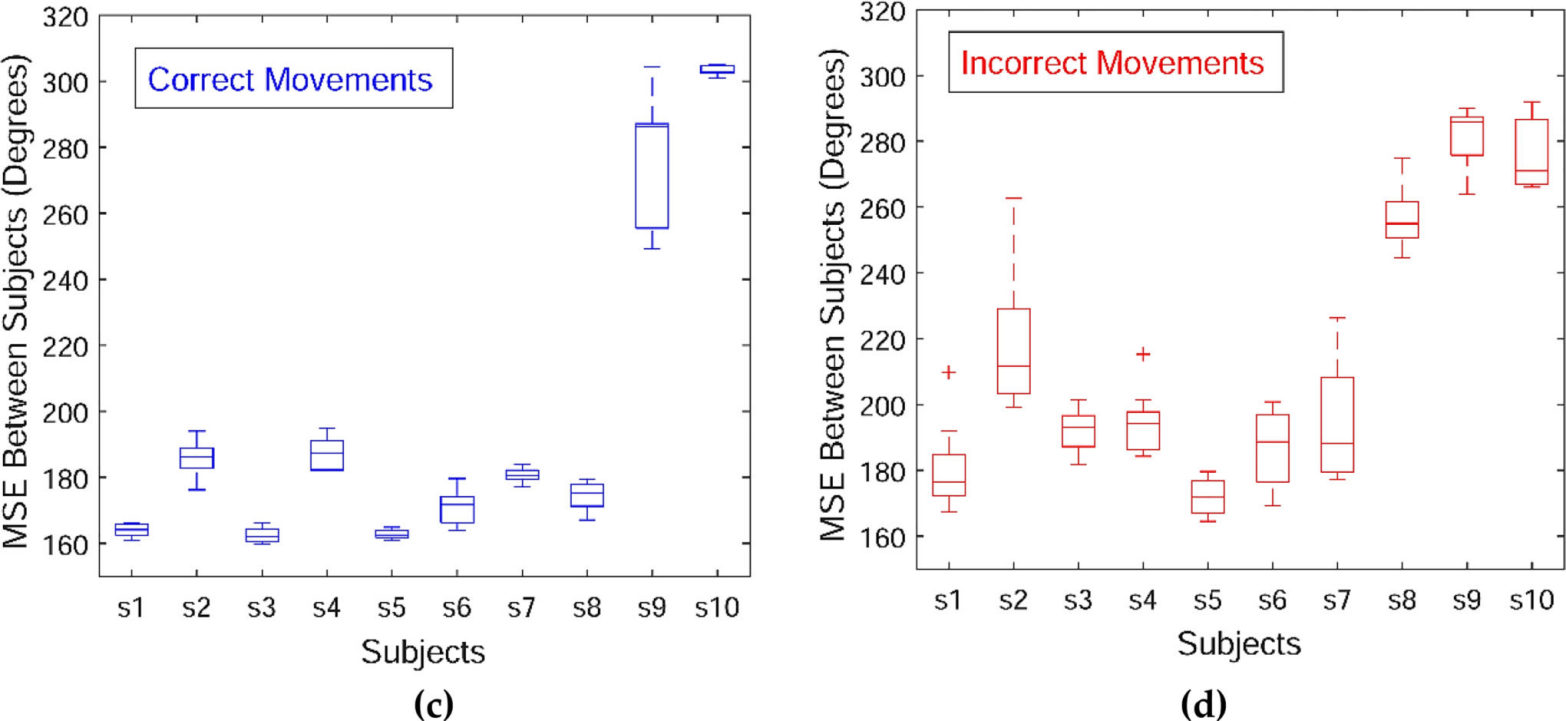
Mean-square deviation for the angular displacements of the deep squat movement across the 10 subjects. **(a)** Within-subjects variance for the correct movements; **(b)** Within-subjects variance for the incorrect movements; **(c)** Between-subjects variance for the correct movements; **(b)** Between-subjects variance for the incorrect movements.

Next, the variability across the movement sequences in the data set is briefly discussed. The skeletal angular data acquired with the Vicon sensory system for the deep squat exercise, i.e., m01 movement, is employed for this purpose. Mean-square deviation (also known as mean-square error, or MSE) is selected as a statistic for comparing the variability in performance within and between subjects. For calculation of the MSE, the movement episodes were first scaled via cubic interpolation to a common length equal to the average number of time steps for all 100 episodes of m01, which was equal to 240. Afterward, the MSE was calculated and it was normalized by dividing the total deviation with the number of time steps (i.e., 240) and the number of dimensions (117 for the Vicon system). The results are shown with box diagrams in Figure 5. The subfigure (a) presents the deviation of the correct movements within-subjects. Most of the subjects were consistent in their performance, except for Subject 9 who completed the repetitions of the dep squat exercise in a less uniform manner. The subfigure (b) displays the deviation within-subjects of the incorrect sequences with respect to the correct movements performed by the same subject. Similarly, the last 3 subjects produced larger variability between their correct and incorrect movements. Analogously, subfigure (c) depicts the variation between-subjects, where the deviation of each correct movements is calculated with respect to all correct movements collected from all 10 subjects. Beside Subject 9, Subject 10 movements are also characterized with a greater disparity relative to the other subjects, although the subject performed the repetitions in a consistent manner based on the Subfigure (a). The subfigure (d) shows the deviation of each incorrect movement with respect to all correct movements.

Table 5 contains the means and standard deviations for the between-subjects MSE deviations of the angular Vicon measurements for all 10 movements. For each movement two rows are provided corresponding to the correct movements (denoted with a ‘-c’ suffix) and the incorrect movements (denoted with an ‘-i’ suffix). The values for the subjects who used a different hand or leg for a particular movement than the majority of the other subjects (as indicted in Table 4) were not included in the table (and are indicated with NA in the table), because their movement data deviates significantly from the other movements. Our intent is to provide a preliminary statistical information on the variability of the movement data, and as part of our future work we will investigate other metrics for explanation of the consistency of the subjects’ performance.

The research project related to the data collection was approved by the Institutional Review Boards at the University of Idaho on April 26, 2017 under the identification code IRB 16–124. A written informed consent for participation in a research study was approved by the board, and was obtained from all participants in the study.

## 4. Conclusion

In summary, the contribution of the paper is the presented data set of movements related to physical therapy exercises. The set of 10 exercises performed by 10 healthy subjects and recorded with two motion capturing systems is described. Instances of the movements performed in an incorrect manner are also provided, and can potentially be utilized for evaluation of data modeling methods. The presented data set have several limitations. In particular, all the movements were performed by healthy subjects; it would have been preferred at least the incorrect movements to have been performed by patients. In addition, the selected movements are general, and are not associated with a particular condition, or groups of patients. A shortcoming of the data set for the purpose of mathematical modeling of the movement data with machine learning methods is its size, whereas a large data set including a large number of subjects and movement types is preferred.

## Figures and Tables

**Table 1:** Movement order and a brief description.

Order	Movement	Description	Non-Optimal Movement
m01	Deep squat	Subject bends the knees to descends the body toward the floor with the heels on the floor, the knees aligned over the feet, the upper body remains aligned in the vertical plane	Subject does not maintain upright trunk posture, unable to squat past parallel, demonstrates knee valgus collapse or trunk flexion greater than 30°
m02	Hurdle step	Subject steps over the hurdle, while the hips, knees and ankles of the standing leg remain vertical	Subject does not maintain upright trunk posture, has less than 89° hip flexion, does not maintain femur in neutral position
m03	Inline lunge	Subject takes a step forward and lowers the body toward the floor to make contact with the knee behind the front foot	Subject unable to maintain upright trunk posture, has rear knee reach the floor, or has a lateral deviation in forward step
m04	Side lunge	Subject takes a step to the side and lowers the body toward the floor	Subject displays moderate to significant knee valgus collapse, pelvis drops or rises more than 5°, trunk angle of less than 30°, thigh angle of more than 45°, center of knee is anterior to the toes
m05	Sit to stand	Subject lifts the body from a chair to a standing position	Subject unable to maintain upright trunk posture, pelvis rises 5° or more, uses arms or compensatory motion to stand, unable to maintain balance or shifts weight to one leg, displays moderate to significant knee valgus collapse
m06	Standing active straight leg raise	Subject raises one leg in front of the body while keeping the leg straight and the body vertical	Subject unable to maintain upright trunk posture, pelvis deviates 5° or more, more than 6° of knee flexion and less than 59° of hip flexion
m07	Standing shoulder abduction	Subject raises one arm to the side by a lateral rotation, keeping the elbow and wrist straight	Subject unable to maintain upright trunk posture or head in neutral position, lift arm does not remain in plane of motion, less than 160° of abduction
m08	Standing shoulder extension	Subject extends one arm rearward, keeping the elbow and wrist straight	Subject unable to maintain upright trunk posture or head in neutral position, lift arm does not remain in sagittal plane, less than 45° of extension
m09	Standing shoulder internal-external rotation	Subject bends one elbow to a 90 degree angle, and rotates the forearm forward and backward	Subject unable to maintain upright trunk posture or head in neutral position, arm positioning less than 60° of motion in both directions
m10	Standing shoulder scaption	Subject raises one arm in front of the chest until reaching the shoulders height, keeping the elbow and wrist straight	Subject unable to maintain upright trunk posture or head in neutral position, lift arm is not maintained in correct plane, less than 90° of motion

**Table 2:** Order of positions and angles in the data set for the Vicon and Kinect systems.

Joint order	Vicon Positions	Vicon Angles	Kinect Positions and Angles
1	LFHD - Left head front	Head (absolute)	Waist (absolute)
2	RFHD - Right head front	Left head	Spine
3	LBHD – Left back head	Right head	Chest
4	RBHD - Rright back head	Left neck	Neck
5	C7 - 7th cervical vertebra	Right neck	Head
6	T10 - 10th thoracic vertabra	Left clavicle	Head tip
7	CLAV - Clavicle	Right clavicle	Left Collar
8	STRN - Sternum	Thorax (absolute)	Left Upper arm
9	RBAK – Right back	Left thorax	Left for arm
10	LSHO - Left shoulder	Right thorax	Left hand
11	LUPA - Left upper arm	Pelvis (absolute)	Right collar
12	LELB - Left elbow	Left pelvis	Right upper arm
13	LFRM - Left forearm	Right pelvis	Right forearm
14	LWRA - Left wrist A	Left hip	Right hand
15	LWRB - Left wrist B	Right hip	Left upper leg
16	LFIN - Left finger	Left femur	Left lower leg
17	RSHO - Right shoulder	Right femur	Left foot
18	RUPA - Right upper arm	Left knee	Left leg toes
19	RELB - Right elbow	Right knee	Right upper leg
20	RFRM - Right forearm	Left tibia	Right lower leg
21	RWRA - Right wrist A	Right tibia	Right foot
22	RWRB - Right wrist B	Left ankle	Right leg toes
23	RFIN - Right finger	Right ankle	
24	LASI - Left ASIS	Left foot	
25	RASI - Right ASIS	Right foot	
26	LPSI - Left PSIS	Left toe	
27	RPSI - Right PSIS	Right toe	
28	LTHI - Left thigh	Left shoulder	
29	LKNE - Left knee	Right shoulder	
30	LTIB - Left tibia	Left elbow	
31	LANK - Left ankle	Right elbow	
32	LHEE - Left heel	Left radius	
33	LTOE - Left toe	Right radius	
34	RTHI - Right thigh	Left wrist	
35	RKNE - Right knee	Right wrist	
36	RTIB - Right tibia	Left upperhand	
37	RANK - Right ankle	Right upperhand	
38	RHEE - Right heel	Left hand	
39	RTOE - Right toe	Right hand	

**Table 3:** Demographic information for the subjects.

Subject ID	Gender	Height (cm)	Weight (kg)	BMI	Dominant side	Profession
s01	Female	169.0	69.4	24.3	Right	Grad student
s02	Male	180.0	83.0	25.6	Right	Grad student
s03	Male	169.5	64.8	22.6	Right	Faculty
s04	Female	178.5	79.4	24.9	Right	Faculty
s05	Male	185.5	148.6	43.2	Right	Grad student
s06	Female	164.6	53.6	19.8	Right	Grad student
s07	Female	166.1	53.1	19.2	Left	Grad student
s08	Male	170.5	77.3	26.6	Right	Grad student
s09	Female	164.0	56.0	20.8	Right	Grad student
s10	Male	174.2	94.7	31.2	Left	Grad student

**Table 4:** Hand or leg used by each subject in performing the 10 movements.

Subject ID	Hurdle step	Inline lunge	Side lunge	Standing ASLR	Standing ShAbd	Standing ShExt	Standing ShIrEr	Standing ShScp
s01	R	L	R	R	R	R	R	R
s02	L	R	R	R	R	R	R	R
s03	R	L	R	R	R	R	R	R
s04	R	L	R	R	R	R	R	R
s05	R	L	R	R	R	R	R	R
s06	R	L	R	R	R	R	R	R
s07	L	R	L	L	L	L	L	L
s08	R	L	R	R	R	R	R	R
s09	R	L	R	R	R	R	R	R
s10	L	R	L	L	L	L	L	L

**Table 5:** Mean-square deviation related to between-subjects measurements of the correct and incorrect movements. The values represent the mean and the standard deviation (in parenthesis) for the MSE per subject. The rows with the values for the correct sequences have a ‘-c’ suffix, and the corresponding rows or the incorrect movements have an ‘-i’ suffix. In addition, the rows for the incorrect movements are shaded. NA values indicate subjects who used a different hand of leg for an exercise than the rest of the subjects.

Subject ID	s01	s02	s03	s04	s05	s06	s07	s08	s09	s10
m01-c	164 (2)	186 (5)	162 (2)	187 (5)	163 (2)	171 (5)	180 (2)	174 (4)	277 (19)	303 (2)
m01-i	180 (13)	219 (21)	192 (6)	194 (9)	171 (6)	187 (12)	194 (16)	257 (9)	281 (10)	276 (11)
m02-c	99 (1)	NA	143 (17)	120 (25)	136 (4)	112 (3)	NA	109 (2)	213 (2)	NA
m02-i	111 (3)	NA	150 (17)	131 (26)	126 (10)	121 (6)	NA	121 (9)	208 (32)	NA
m03-c	161 (1)	NA	NA	166 (3)	175 (7)	196 (26)	NA	218 (22)	163 (1)	NA
m03-i	180 (4)	NA	NA	178 (5)	192 (16)	211 (17)	NA	228 (23)	175 (7)	NA
m04-c	170 (2)	146 (14)	154 (12)	151 (18)	135 (14)	138 (3)	NA	128 (6)	152 (20)	NA
m04-i	267 (3)	199 (31)	163 (26)	147 (4)	168 (16)	144 (2)	NA	138 (9)	162 (19)	NA
m05-c	309 (2)	178 (2)	163 (3)	164 (2)	169 (14)	186 (3)	168 (9)	199 (29)	215 (3)	205 (17)
m05-i	245 (5)	189 (26)	188 (18)	182 (10)	211 (19)	205 (7)	187 (15)	218 (24)	216 (27)	210 (9)
m06-c	231 (1)	179 (21)	206 (46)	192 (5)	185 (5)	174 (2)	NA	196 (8)	226 (23)	NA
m06-i	208 (3)	184 (10)	185 (4)	187 (7)	195 (9)	180 (3)	NA	205 (10)	231 (14)	NA
m07-c	85 (2)	77 (4)	76 (3)	75 (3)	82 (8)	96 (9)	NA	103 (16)	78 (5)	NA
m07-i	99 (7)	104 (9)	108 (10)	115 (9)	104 (10)	155 (62)	NA	207 (48)	123 (15)	NA
m08-c	127 (1)	115 (5)	105 (4)	100 (2)	105 (7)	117 (1)	NA	169 (17)	129 (38)	NA
m08-i	183 (33)	120 (8)	113 (6)	108 (5)	118 (10)	128 (4)	NA	123 (2)	122 (6)	NA
m09-c	223 (2)	126 (40)	111 (2)	111 (3)	115 (3)	123 (5)	NA	188 (25)	145 (41)	NA
m09-i	138 (8)	120 (7)	121 (8)	127 (7)	159 (39)	142 (13)	NA	129 (6)	124 (12)	NA
m10-c	133 (1)	127 (9)	129 (5)	128 (3)	141 (47)	293 (16)	NA	197 (36)	195 (8)	NA
m10-i	204 (2)	141 (24)	137 (3)	140 (6)	157 (43)	269 (10)	NA	199 (24)	161 (25)	NA
